# Knowledge translation strategies to support the sustainability of evidence-based interventions in healthcare: a scoping review

**DOI:** 10.1186/s13012-023-01320-0

**Published:** 2023-12-04

**Authors:** Rachel Flynn, Christine Cassidy, Lauren Dobson, Joyce Al-Rassi, Jodi Langley, Jennifer Swindle, Ian D. Graham, Shannon D. Scott

**Affiliations:** 1 School of Nursing and Midwifery, Brookfield Health Sciences Complex, University College of Cork, College Road Cork, Cork, T12 AK54 Ireland; 2 Faculty of Nursing, Level 3, Edmonton Clinic Health Academy, University of Alberta, 11405 87 Avenue, Edmonton, Alberta T6G 1C9 Canada; 3 School of Nursing, Faculty of Health, Dalhousie University, Room N21, Forrest Bldg., PO Box 15000 5869 University Avenue Halifax, Nova Scotia, B3H 4R2 Canada; 4 School of Epidemiology and Public Health, University of Ottawa, 600 Peter Morand Crescent, Ottawa, ON K1G 5Z3 Canada; 5 The Centre for Implementation Research, Ottawa Hospital Research Institute, 501 Smyth Road, Box 241, Ottawa, Ontario K1H 8L6 Canada

**Keywords:** Knowledge translation strategies, Sustainability, Evidence-based interventions, Implementation, Healthcare

## Abstract

**Background:**

Knowledge translation (KT) strategies are widely used to facilitate the implementation of EBIs into healthcare practices. However, it is unknown what and how KT strategies are used to facilitate the sustainability of EBIs in institutional healthcare settings.

**Objectives:**

This scoping review aimed to consolidate the current evidence on (i) what and how KT strategies are being used for the sustainability of EBIs in institutional healthcare settings; (ii) the reported KT strategy outcomes (e.g., acceptability) for EBI sustainability, and (iii) the reported EBI sustainability outcomes (e.g., EBI activities or component of the intervention continue).

**Methods:**

We conducted a scoping review of five electronic databases. We included studies describing the use of specific KT strategies to facilitate the sustainability of EBIs (more than 1-year post-implementation). We coded KT strategies using the clustered ERIC taxonomy and AIMD framework, we coded KT strategy outcomes using Tierney et al.’s measures, and EBI sustainability outcomes using Scheirer and Dearing’s and Lennox’s taxonomy. We conducted descriptive numerical summaries and a narrative synthesis to analyze the results.

**Results:**

The search identified 3776 studies for review. Following the screening, 25 studies (reported in 27 papers due to two companion reports) met the final inclusion criteria. Most studies used multi-component KT strategies for EBI sustainability (*n* = 24). The most common ERIC KT strategy clusters were to train and educate stakeholders (*n* = 38) and develop stakeholder interrelationships (*n* = 34). Education was the most widely used KT strategy (*n* = 17). Many studies (*n* = 11) did not clearly report whether they used different or the same KT strategies between EBI implementation and sustainability. Seven studies adapted KT strategies from implementation to sustainability efforts. Only two studies reported using a new KT strategy for EBI sustainability. The most reported KT strategy outcomes were acceptability (*n* = 10), sustainability (*n* = 5); and adoption (*n* = 4). The most commonly measured EBI sustainability outcome was the continuation of EBI activities or components (*n* = 23), followed by continued benefits for patients, staff, and stakeholders (*n* = 22).

**Conclusions:**

Our review provides insight into a conceptual problem where initial EBI implementation and sustainability are considered as two discrete time periods. Our findings show we need to consider EBI implementation and sustainability as a continuum and design and select KT strategies with this in mind. Our review has emphasized areas that require further research (e.g., KT strategy adaptation for EBI sustainability). To advance understanding of *how* to employ KT strategies for EBI sustainability, we recommend clearly reporting the dose, frequency, adaptations, fidelity, and cost of KT strategies. Advancing our understanding in this area would facilitate better design, selection, tailored, and adapted use of KT strategies for EBI sustainability, thereby contributing to improved patient, provider, and health system outcomes.

**Supplementary Information:**

The online version contains supplementary material available at 10.1186/s13012-023-01320-0.

Contributions to the literature • Sustainability is concerned with the continued use and benefit of effective evidence-based interventions.

• Knowledge translation (KT) strategies are widely used to facilitate the implementation of evidence-based interventions in healthcare.

• However, there is no comprehensive evidence on what and how KT strategies are used to facilitate the sustainability of EBIs across institutional healthcare settings.

• This review provides consolidated evidence of the most reported KT strategies for the sustainability of EBIs (train and educate stakeholders), the most reported KT strategy outcomes of KT strategies used for EBI sustainability (acceptance), and the most reported sustainability outcomes of EBIs in institutional healthcare settings (continuation of EBI components).

• The results can be used to guide the planning, design, and selection of the KT strategies for the sustainability of EBIs in healthcare settings. It also highlights where additional research is warranted on KT strategies for EBI sustainability.

## Introduction

Evidence-based interventions (EBIs) are innovations, practices, programs, or policies with proven efficacy and effectiveness [1]. Despite enormous investments in the development of EBIs for healthcare improvement, 60% of care provided is aligned with the best evidence, 30% of care is wasteful or inappropriate and 10% is harmful [2, 3]. Furthermore, only 23% of EBIs are sustained 2 years after initial implementation, leading to unnecessary healthcare waste and reduced benefits for patients, providers, and systems [4].

To address these gaps, knowledge translation (KT) focuses on the application of innovation to practice or policy to improve patients’ health outcomes and strengthen the healthcare system with more effective health services and EBIs [5]. KT occurs through a dynamic and iterative process of knowledge synthesis, dissemination, and exchange between researchers, decision-makers, and knowledge users [6]. A central goal of KT is to ensure that stakeholders are aware of and use research evidence to inform their health and healthcare decision-making. Stakeholders for KT, include policymakers, professionals, patients, family members, informal carers, researchers, and industry [7]. *KT strategies* are approaches designed to promote the use of EBIs in healthcare practices and policy and to help close research-practice gaps (i.e., what we know versus what we do) [7]. KT strategies can be single or multifaceted in nature, target multiple levels (individual, team, system), and focus on the implementation, adoption, and/or sustainability of an EBI [8, 9].

Taxonomies such as the Expert Recommendations for Implementing Change (ERIC) [10, 11] have been developed to establish a common classification system for KT strategy terms, definitions, and categories. Examples of KT strategies include (1) audit and feedback: where data is summarized about specific aspects of practice and provided to practitioners to encourage practice change [12–16], (2) facilitation: where an internal and/or external person acts as enabler for the process of implementation [12], and (3) educational outreach: where a trained person or expert visits practice settings and provides information, such as new evidence to change practice [16]. There is an abundance of evidence on the varying degrees of effectiveness of different types of KT strategies [15, 17–19] used to facilitate the implementation of various EBIs with different stakeholders such as nurses [20, 21], physicians [22, 23], and allied health [24] for various health conditions [25, 26] across different health contexts [27, 28]. Recent synthesis efforts have identified KT strategies used to facilitate the sustainability (long-term use and benefits) of EBIs in public health [29, 30], and for chronic diseases [31]. Most recently a synthesis on the efficacy of sustained knowledge translation (KT) strategies in chronic disease management found that over the long term, continuing to use KT strategies was rarely defined and infrequently assessed, suggesting fundamental gaps in knowledge [32]. There is no synthesized and consolidated empirical evidence on what and how KT strategies are used to facilitate the sustainability of EBIs in institutional healthcare settings (e.g., hospital organizations and long-term care facilities). Given that most of the healthcare EBIs are implemented in institutional healthcare settings it is important to understand, what and how KT strategies facilitate EBI sustainability in these contexts.

Sustainability is a priority issue for health services research and has been described as “one of the most significant translational research problems of our time” 33 (pg. 89). Over the last decade, less than 1% of research has focused on the sustainability of EBIs for healthcare [4, 33, 34]. One challenge with sustainability research has been the variation in its conceptualization resulting in operationalization and measurement challenges. As a result, Moore et al. developed a comprehensive definition that states that sustainability occurs when an EBI continues to be delivered and maintained after a defined time, during which the EBI and individual and collective behavior change may evolve or adapt while continuing to produce benefits for individuals/systems [35] (pg. 114). Our review is guided by this multicomponent definition of sustainability.

It is unclear if there are similarities and differences between KT strategies used to facilitate implementation (initial use of EBIs) and strategies used to facilitate the sustainability (ongoing use) of EBIs for healthcare. It is also unknown if the same KT strategies can support both implementation and sustainability or if specific KT sustainability strategies need to be developed and used. There is a need for this evidence to inform the selection, design, and use of KT strategies to facilitate the ongoing use of EBIs in institutional healthcare settings.

This scoping review aimed to consolidate the current evidence on (i) what and how KT strategies are being used for the sustainability of different EBIs across various institutional healthcare settings; (ii) what KT strategy outcomes (i.e., feasibility, acceptability, appropriateness, fidelity, adoption, cost) are reported on for the sustainability of EBIs in institutional healthcare settings; and (iii) what EBI sustainability outcomes (i.e., continuation of benefits for patients) are reported in the current evidence base. The rationale for looking at both KT strategy outcomes (e.g., the feasibility of external facilitators for the sustainability of an EBI) and EBI sustainability outcomes (e.g., the continuation of benefits for patients, staff, and stakeholders as a result of the EBI) is to understand how acceptable, feasible, etc. the KT strategy was or not for supporting EBI sustainability and whether the EBI itself continued to produce improved outcomes for patients, practices, and policy. A scoping review was deemed to be the appropriate method as it allowed for examination of the “extent, range and nature of research activity” in the use of KT strategies for the sustainability of EBIs in institutional healthcare settings (i.e., hospital organizations and long-term care settings) [36]. The key terms used for this review are presented in Table 1.

**Table 1:** Review terms

Term	Definition
Evidence-based interventions (EBIs)	Innovations, practices, programs, or policies with proven efficacy and effectiveness [1].
Implementation	The process of integrating evidence-based interventions into a specific setting [37].
Knowledge translation	“Dynamic and iterative process that includes the synthesis, dissemination, exchange and ethically sound application of knowledge to improve health, provide more effective health services and products and strengthen the health care system.*”* [5]
Knowledge translation strategies	*A*pproaches designed to promote the use of EBIs in healthcare practices and policy and to help close research-practice gaps (i.e., what we know versus what we do) [7, 38]. Also known as implementation strategies, implementation interventions, or KT interventions.
Adoption	“The intention, initial decision, or action to try or employ an innovation or evidence-based practice. Adoption also may be referred to as “uptake.” [39].
Sustainment	“The sustained use or delivery of an intervention in practice following cessation of external implementation support.” [40].
Sustainability	“Occurs after a defined period of time, the program, clinical intervention, and/or implementation strategies continue to be delivered and/or individual behavior change (i.e., clinician, patient) is maintained; the program and individual behavior change may evolve or adapt while continuing to produce benefits for individuals/systems.” [35] (pg2.). We used the term “sustainability” to refer to both the desired outcome and the characteristics or processes by which the EBI is more likely maintained.
KT strategy outcomes (defined by Proctor et al. as implementation outcomes)	“The effects of deliberate and purposive actions to implement new treatments, practices, and services [39]. (pg. 65) ^“^They serve as indicators of the implementation success. Second, they are proximal indicators of implementation processes. And third, they are key intermediate outcomes.” [39]
Sustainability outcomes	“The subsequent impact (healthcare improvement or public health outcomes) of sustained intervention use” [41]

## Research aims and context

The following research questions guided this review:

1. *What KT strategies* have been used to facilitate the sustainability of EBIs in institutional healthcare settings within peer-reviewed publications?
2. *How have KT strategies* been used to facilitate the sustainability of EBIs in institutional healthcare settings?
3. What *KT strategy outcomes* are reported in the included studies?
4. What *sustainability outcomes of the EBI* are reported in the included studies?

## Methods

We conducted this scoping review using the Arksey and O’Malley framework [36] for conducting scoping reviews and the Joanna Briggs Institute Methodology for Scoping Reviews [42]. The Preferred Reporting Items for Systematic Reviews and Meta-Analysis (PRISMA) flow chart [43] and the PRISMA for Searching (PRISMA-S) extension (Additional file 1) [44] to guide the reporting of our scoping review. There is no published protocol for this review.

To identify relevant studies, we developed inclusion and exclusion criteria based on the population, concept, and context mnemonic recommended by the Joanna Briggs Institute Methodology for Scoping Reviews [42] (Table 2).

**Table 2:** Review inclusion and exclusion criteria

	Inclusion Criteria	Exclusion Criteria
*Participants*	Studies that report on the sustainability of EBIs at multiple levels, including individual, team, and organizational levels.	Studies of participants not in healthcare.
*Concept*	Studies that report and provide a description of the use of specific KT strategies to facilitate the sustainability of EBIs. We employed the following operational definitions: KT strategies promote the translation of research evidence into practice [7]. Evidence-based interventions are defined as treatments, practices, programs, policies, or guidelines with proven efficacy and effectiveness [1]. Sustainability occurs after a defined period of time, where the EBI continues in practice, KT strategies continue to be delivered, behavior change is maintained, and where the EBI and behavior change may evolve or adapt while continuing to produce benefits for individuals/systems [35].	No explicit description of KT strategy. No explicit description of EBI. EBI did not extend beyond 1 year of initial implementation or did not look at sustainability after the termination of research funds. Studies that contain both implementation and sustainability descriptions but do not explicitly provide a detailed breakdown of related sustainability efforts. Studies that ONLY evaluate determinants (barriers and facilitators) of sustainability (not related to the KT strategy described).
*Context*	Studies focused on the sustainability of EBIs in healthcare institutional settings (hospital organizations and long-term care settings)	Studies focused on public health, primary care, and family medicine/general practice. Studies conducted outside a healthcare context (e.g., schools, social care settings).
*Types of Studies*	Primary, peer-reviewed research studies- not limited by research design. Studies published in English. Studies published from database inception to present.	Grey literature sources. Secondary research that did not include original data. Literature reviews.

### Search methods

The search methods for this review are in adherence to the PRISMA for Searching (PRISMA-S) extension [44] An experienced health sciences librarian (MK) conducted comprehensive systematic searches in November 2021. She searched the following databases from inception to November 3rd, 2021: Medline and EMBASE via OVID; Cumulative Index to Nursing and Allied Health Literature (CINAHL) via EBSCOhost; Scopus via Elsevier; Cochrane Library via Wiley. In consultation with the research team, MK created a robust search strategy derived from three main concepts: (1) program or initiative sustainability, including long-term changes or continuous adoption; (2) knowledge translation or transfer, quality improvement, organizational change, implementation, implementation science, diffusion of innovation, and other related knowledge translation terminology; (3) evidence-based, evidence-informed, or evidence-supported practice, including searching for clinical pathways and healthcare policies. Databases were searched using a combination of natural language terms (keywords) and controlled terms (subject headings, e.g., MeSH), wherever they were available. No language or publication date limits were applied. Study type limits were not applied but items such as news reports, opinion pieces, editorials, notes, and conference materials were removed from the results. We also hand-searched the reference lists of included papers to identify additional records. See Additional file 2 for the full search strategy by database.

### Study selection

We exported the search results from each database in complete batches and imported them to the systematic review management software, Covidence [45] to identify and remove duplicate records and to facilitate title/abstract and full-text screening. Overall, we identified 7704 records through database searches and removed 3846 records as duplicates, leaving 3858 records for title/abstract screening. Two independent reviewers (LD and JAR) screened titles and abstracts for assessment against the inclusion and exclusion criteria. We only included studies that reported on an EBI defined as a practice, program, innovation, or policy that had been established as effective through previous research. Two independent reviewers (LD and JAR) assessed the full text of selected studies in detail against the inclusion and exclusion criteria. We recorded the reasons for exclusion directly in Covidence. We resolved any disagreements between the reviewers at each stage of the selection process through discussion, or by a third and fourth reviewer (CC and RF).

### Data extraction and analysis

We used a data extraction tool developed by the research team to extract data from the included studies, using Microsoft Excel. We initially piloted the data extraction tool with five studies and modified it as needed. Two reviewers independently extracted (LD, JL) the following study information: author(s); year of publication; country of origin; study aim/purpose; study discipline and; level of analysis (individual, team or organizational); study setting; reporting of sex/gender; design and length of study; description of evidence-based intervention; description of KT strategy; the aims, ingredients, mechanism, and delivery of the KT strategies; description of KT strategy outcomes, description of EBI sustainability outcomes. A third and fourth reviewers resolved any questions or discrepancies (CC, RF).

Two reviewers (LD, JL) used several frameworks and taxonomies to chart and analyze the extracted data advised by (RF, CC, IDG, and SDS) (see Table 3). We used the AIMD framework [47] to extract details about the KT strategy, including the aim, ingredients, mechanisms, and delivery. Next, we used the Clustered ERIC taxonomy [10] to classify the KT strategies by the following nine clusters: (1) use of evaluative and iterative strategies; (2) provide interactive assistance; (3) adapt and tailor to context; (4) develop stakeholder interrelationships; (5) train and educate stakeholders; (6) support clinicians; (7) engage consumers; (8) utilize financial strategies; (9) change infrastructure. We used Tierney et al.’s [49] 10 implementation measures recommended to extract when conducting evidence syntheses to extract and report the KT strategy outcomes used in the included studies: (1) acceptability, (2) adoption, (3) appropriateness, (4) feasibility, (5) fidelity, (6) implementation cost, (7) intervention complexity, (8) penetration, (9) reach, and (10) sustainability of KT strategies [NO_PRINTED_FORM]. We used Scheirer and Dearing’s taxonomy (expanded by Lennox et al.) [34, 48] to extract and report the sustainability outcomes of the EBI: (1) Benefits for patients, staff and stakeholders continue, (2) activities or components of the EBI continue, (3) maintenance of relationships, partnerships, or networks, (4) maintenance of new procedures, and policies, (5) attention and awareness of the problem or issue are continued or increased, (6) replication, roll-out or scale-up of the EBI, (7) capacity built within staff, stakeholders and communities continues, (8) adaptation of the EBI in response to new evidence or contextual influences and (9) gaining further funds to continue the EBI and maintain improvements.

**Table 3:** Data extraction frameworks

Review question	Data extraction category	Tool or framework	Description
1. What KT strategies have been used to facilitate the sustainability of EBIs in institutional healthcare settings within peer-reviewed publications?	KT strategy description	Clustered ERIC taxonomy [46]	The ERIC taxonomy consists of 73 distinct knowledge translation with definitions, which has been used to understand the operationalization of implementation strategies [32]. Waltz and colleagues conducted a categorization and strategy ratings of importance and feasibility for the strategies and put them into 9 broad clusters, which will be used for this review: (1) use of evaluative and iterative strategies; (2) provide interactive assistance; (3) adapt and tailor to context; (4) develop stakeholder interrelationships; (5) train and educate stakeholders; (6) support clinicians; (7) engage consumers; (8) utilize financial strategies; (9) change infrastructure. We coded the KT strategies based on descriptions of these 9 broad categories.
2. How have KT strategies been used to facilitate the sustainability of EBIs in institutional healthcare settings?	KT strategy description	AIMD Framework [47]	(1) Aims: what do you want your intervention to achieve and for whom? (2) Ingredients: what comprises the intervention? (3) Mechanisms: how do you propose the intervention will work? and (4) Delivery: how will you deliver the intervention?
3. What KT strategy outcomes are reported in the included studies?	KT strategy outcomes	10 implementation outcomes [39]	Acceptability, adoption, appropriateness, feasibility, fidelity, implementation cost, intervention complexity, penetration, reach, and sustainability [39].
4. What sustainability outcomes of the EBI are reported in the included studies?	Sustainability outcomes of the EBI	9 categories of sustainability outcomes [34, 48]	1. Benefits for patients, staff, and stakeholders continue, 2. EBI activities or components of the intervention continue, 3. Maintenance of relationships, partnerships, or networks, 4. Maintenance of new procedures, and policies, 5. Attention and awareness of the problem or issue is continued or increased and 6. Replication, roll-out, or scale-up of the EBI, 7. Capacity built within staff, stakeholders, and communities continues, 8. Adaptation of the EBI in response to new evidence or contextual influences and 9. Gaining further funds to continue the EBI and maintain improvements

Further, we extracted additional information on the reported KT strategies, including (1) whether the same KT strategies were used for implementation and sustainability, (2) new KT strategies were introduced for sustainability, or (3) adaptations were made to the KT strategies to support sustainability of the EBI. We produced descriptive numerical summaries of the quantitative data (i.e., frequency of KT strategy, barriers and facilitators, and outcomes). Next, we conducted deductive content analysis to categorize qualitative data into the respective frameworks and taxonomies and reported these narratively and in tabular formats [50].

## Results

**Fig. 1:**
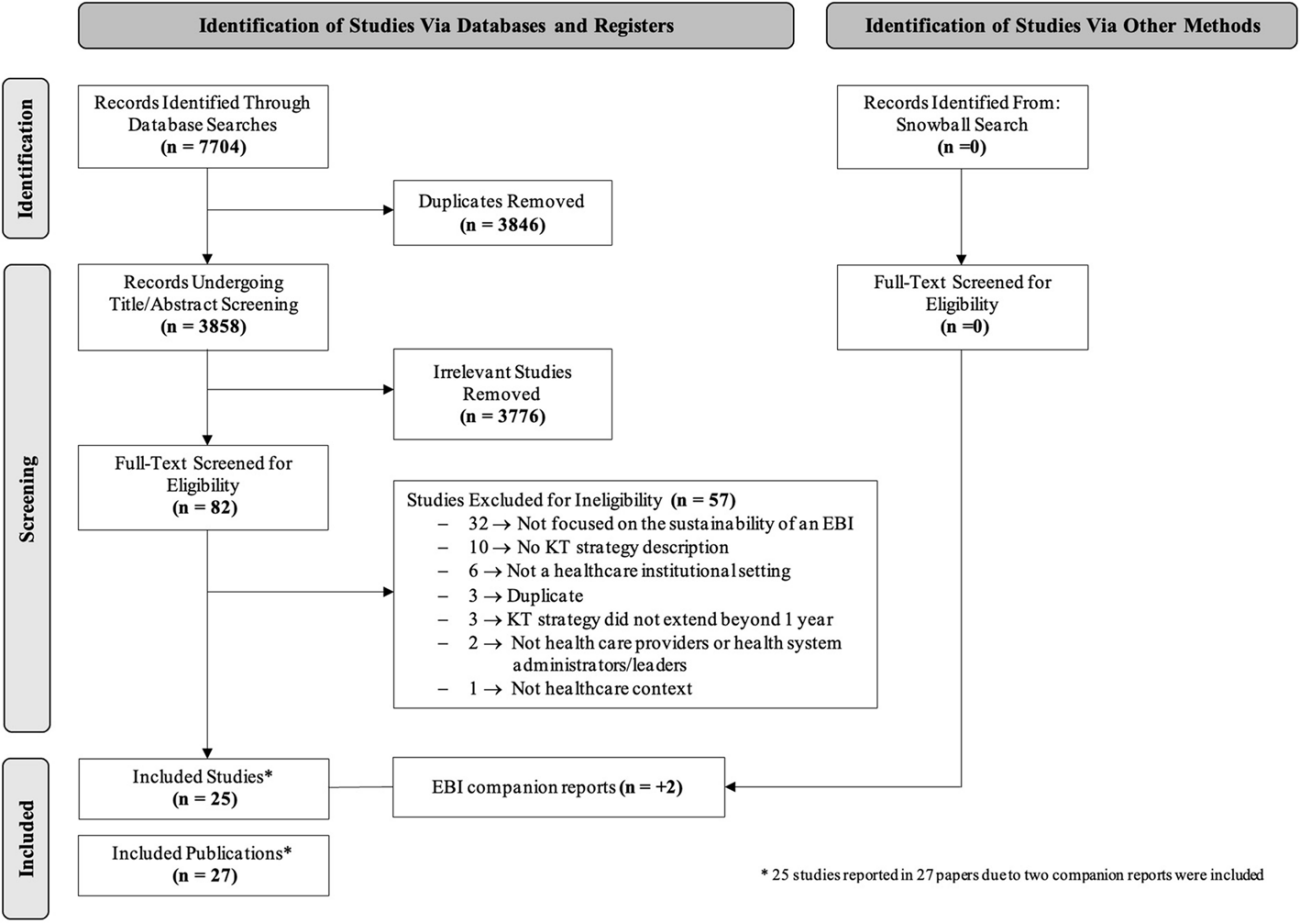
PRISMA flow diagram of included studies

After the removal of duplicates, we screened 3858 titles and abstracts against the inclusion criteria. 82 studies were included for full-text screening. From this set, we excluded 57 studies that did not meet the inclusion criteria. In total, 25 studies (reported in 27 papers due to 2 companion reports) met the final inclusion criteria for the review (Fig. 1) [51–77].

### Study characteristics

**Fig. 2:**
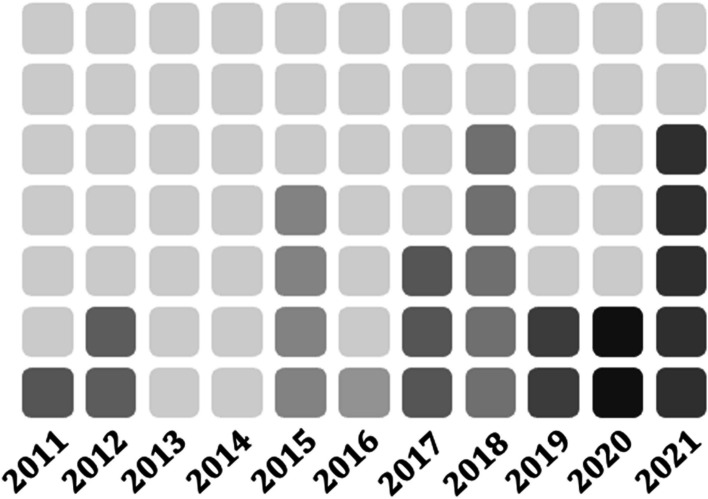
Year of publication of included studies

Study characteristic details are described in Table 4. All 25 studies were published between 2011 and 2021 (Fig. 2). The 25 included studies reported on 16 health disciplines. Most studies focused on the adult population (*n* = 16) [51, 55–57, 59, 61, 62, 64, 66–71, 73, 74], while fewer had a pediatric focus (*n* = 8) [53, 58, 60, 63, 65, 72, 75, 77], and one reported on the neonatal intensive care unit [76]. Thirteen of the included studies took place in acute care [55, 57, 60–62, 66, 68, 69, 71, 73, 75–77], 11 in tertiary care [51, 53, 56, 58, 59, 63–65, 70, 72, 74], and 1 in ambulatory care [67]. Fifty-two percent (*n* = 13) [53, 56–58, 61–63, 65, 67, 71, 75–77] of the included studies were published in the USA, with the remaining (*n* = 12) published in Australia (*n* = 2) [68, 74], Canada (*n* = 2) [60, 69], Italy (*n* = 2) [66, 72], Sweden (*n* = 2) [59, 73], Spain (*n* = 1) [70], United Kingdom (*n* = 1) [64], Netherland (*n* = 1) [51], and Northern Ireland (*n* = 1) [55]. Mixed method research was the most common study design (*n* = 8) [51, 57–59, 67, 71, 74, 75]. Other study designs included quantitative descriptive (*n* = 8) [53, 56, 63, 65, 66, 68, 70, 76], quantitative non-randomized (*n* = 4) [62, 69, 72, 73], qualitative (*n* = 3) [55, 64, 77], and cluster randomized control trials (*n* = 2) [60, 61]. While many studies did not explicitly define sustainability (*n* = 11) [53, 57, 58, 63, 64, 69–73, 76], all included studies reported on sustainability of an EBI from 1 to 10 years post-implementation, with 5–9 years being the most commonly reported timeframe (*n* = 8) [51, 53, 56, 62, 66, 68, 74, 76], and a median reported timeframe of 4 years. The most common types of EBIs were practice guidelines (*n* = 12) and care pathways (*n* = 8).

**Table 4:** Study characteristics

Author	Country	Setting	Study discipline	EBI
Algaze, 2018 [75]	USA	Acute	Pediatric Cardiology	Liverpool care pathway
Allen, 2018 [74]	Australia	Tertiary	Nursing	Pain management best practices
Andersson, 2017 [73]	Sweden	Acute	General	Pain Management & analgesic Best Practice Guidelines (BPG)
Barbieri, 2020 [72]	Italy	Tertiary	Pediatrics, Pharmacology, Emergency	Antibiotic stewardship pathway
Becker, 2021 [71]	USA	Acute	Nursing	Bedside shift report
Comino-Sanz, 2018 [70]	Spain	Tertiary	General, Neurology	Fall prevention and management protocol
Erdei, 2015 [76]	USA	Acute	Neonatal Intensive Care	Multi-component intervention for elimination of CLABSI
Jaladanki, 2021 [77]	USA	Acute	Pediatrics	Pediatric asthma pathway
Kingsnorth, 2015 [69]	Canada	Acute	Nursing	Pain BPG
*Lai, 2012* [68]	Australia	Acute	Orthopedics	Osteoporosis pathway
Macdonald, 2021 [67]	USA	Ambulatory	PT	Proactive physical therapy program
McConnell, 2015 [55]	Northern Ireland	Acute	General, Palliative	Liverpool care pathway
Moro, 2017 [66]	Italy	Acute	General	Hand hygiene BPG
Nkoy, 2015 [53]	USA	Tertiary	Pediatric Respirology	Asthma BPG
Oetgen, 2018 [65]	USA	Tertiary	Surgical, Pediatrics	Care pathway for post-op spinal fusion
Parand, 2012 [64]	UK	Tertiary	General	UK Safer Patients Initiative (SPI)
Rutman, 2017 [63]	USA	Tertiary	Pediatrics, Gastro, Emergency	Care pathway for acute gastroenteritis (AGE)
Santos, 2019 [62]	USA	Acute	Obstetrics	Protocol for shoulder dystocia and abnormal fetal heart tracings
Schnipper, 2021 [57]	USA	Acute	Nursing	Toolkit for medication history and reconciliation
Mixon, 2019 [52]				
Shuman, 2018 [61]	USA	Acute	General	Pain management post hip fracture pathway
Titler, 2009 [54]				
Stevens, 2016 [60]	Canada	Acute	General, Pediatrics	Pain management and assessment
Storm-Versloot, 2011 [51]	Netherlands	Tertiary	Surgery	BPG to stop taking routine temperature measurements
Sving, 2020 [59]	Sweden	Tertiary	Nursing, PT/OT, Dietetics	Pressure ulcer prevention program
Tamboli 2020 [56]	USA	Tertiary	Anesthesiology	Total hip/knee arthroplasty clinical pathway
Willis, 2019 [58]	USA	Tertiary	Pediatrics, Cardiology, Surgery	Clinical pathway for pediatric congenital heart disease patients

### KT strategies to facilitate the sustainability of EBIs

Table 5 reports on the KT strategies used to facilitate sustainability and an additional file describes the reported aims, ingredients, mechanism, and delivery of the KT strategies [see Additional file 3]. Among the 25 studies, 66 KT strategies were used. Most studies employed multi-component KT strategies and only one study reported a single-component KT strategy (education) [65]. The three most common KT strategy clusters, as per the ERIC taxonomy [10] were training and educating stakeholders (*n* = 38), developing stakeholder interrelationships (*n* = 33), and the use of evaluative and iterative strategies (*n* = 29). Under the ERIC KT strategy cluster of train and educating stakeholders (*n* = 38), formal education such as seminars and modules were the most common modes of delivery for training and educating staff (*n* = 19) with the aim of increasing awareness on the EBI. Other popular education-related KT strategies were delivered via pocket cards (*n* = 3) and EBI-specific toolkits (*n* = 2). Under the ERIC KT strategy cluster of the development of stakeholder interrelationships (*n* = 33), EBI champions (*n* = 13) and multidisciplinary teams (*n* = 7) were the most common KT strategies used for the sustainability of the EBI, while under the cluster of evaluative and iterative KT strategy, audit and feedback (*n* = 16) were the most frequently reported. Other KT strategies used for the sustainability of the EBI were clinician support, specifically leadership support (*n* = 9), ongoing reminders (*n* = 6), and EBI facilitators (*n* = 5).

**Table 5:** KT strategies used for the sustainability of EBIs

ERIC KT strategy cluster	Examples of KT strategy	
Evaluative and iterative strategies	- Audit and FEEDBack	*n* = 15
	-Tests of change	*n* = 3
	- Teleconference	*n* = 2
	- Quality monitoring	*n* = 2
	- Implementation manual	*n* = 1
	- Return on investment calc	*n* = 1
	- Development of standard tool	*n* = 1
	- Training scorecard	*n* = 1
	- Focus groups	*n* = 1
Interactive assistance	- Facilitators	*n* = 4
	- First line managers	*n* = 1
	- Data experts	*n* = 1
Adapt and tailor to context	- Integration of aims	*n =* 2
	- EHR revisions	*n =* 2
	- Integration of workflow	*n =* 1
	- Knowledge broker	*n =* 1
	- System change	*n =* 1
	- Integrate with standards of care	*n =* 1
	- Revised documentation tool	*n =* 1
Develop stakeholder interrelationships	- EBI champion	*n =* 13
	- Multidisciplinary team	*n =* 6
	- Champion meetings	*n =* 1
	- Implementation team	*n =* 1
	- Knowledge sharing	*n =* 1
	- Multidisciplinary huddle	*n =* 1
	- Opinion leaders	*n =* 1
	- Outreach visits	*n =* 1
	- Program coordinator	*n =* 1
	- QI committee	*n =* 1
	- Site visits	*n =* 1
	- Summit	*n =* 1
Train and educate stakeholders	- Education	*n =* 19
	- Pocket cards	*n =* 3
	- Training	*n =* 3
	- Toolkit	*n =* 2
	- Presentations	*n =* 2
	- Electronic toolkit	*n =* 1
	- Instructional video	*n =* 1
	- Learning collaboratives	*n =* 1
	- Posters	*n =* 1
	- Quick reference guides (QRG)	*n =* 1
	- Summit	*n =* 1
	- Teleconference	*n =* 1
Support clinicians	- Leadership support	*n =* 9
	- Reminders	*n =* 6
	- Increasing capacity	*n =* 1
	- Integration of aims	*n =* 1
	- Mentors	*n =* 1
	- Program manager	*n =* 1
	- Transparent communication	*n =* 1
	- Visits	*n =* 1
Engage consumers	- Provincial Patient and Family Advisory Council (PFAC)	*n =* 2
	- Website	*n =* 2
	- Campaigns	*n =* 1
	- Maintenance of high profile	*n =* 1
	- Patient brochure	*n =* 1
	- Patient/family input	*n =* 1
Utilize financial strategies	- Grant funding	*n =* 1
Change infrastructure	- Creation of virtual tools	*n =* 1

Few studies reported explicit details on how the KT strategy was delivered, including the dose and frequency of the KT strategy (see Additional file 3). For example, Becker et al. and MacDonald et al. described having EBI champions lead monthly meetings with staff [67, 71]. Schnipper et al. reported detailed training activities, including three peer-to-peer webinars and four regional workshops [57]. Other studies provided some detail on how they conducted audit and feedback strategies for EBI sustainability, including how often the audit occurred and how feedback was shared [55, 59, 62, 67, 76]. From a synthesis of the qualitative data across the included studies, ongoing reminders (verbal, visual, and electronic) that were provided by local champions were reported to facilitate the sustainability of the EBI [51, 56, 63, 64, 66, 69, 77]. Furthermore, participants perceived that EBI-related resources built into Electronic Health Record systems and existing structures acted as a consistent reminder for staff to continue the EBI as the standard of care and improved the chances of sustaining lessons learned [69, 77]. One study reported that hiring a designated facilitator was felt to be a key strategy to “ maintaining EBI ‘visibility’, reducing anxiety among nurses, and increasing their confidence regarding the delivery of the EBI” [55] (pg.70). Despite these insights, the majority of included studies lacked specific details on how the KT strategies were used to facilitate the sustainability of the EBI.

Many studies (*n* = 11) did not clearly report whether they used different or the same KT strategies between EBI implementation and EBI sustainability [51, 55, 56, 58, 60, 63, 65, 66, 70, 72, 73]. However, some studies (*n* = 7) reported KT strategies that were adapted from the initial implementation period and used to support the sustainability of the EBI (Table 6). For example, one study changed the frequency of their audit cycles, as outcomes were maintained, audits decreased in frequency from weekly to monthly, then quarterly [71]. Nkoy et al. report adapting their initial KT strategies to fit local needs and integrate them into the workflow [53], and Santos et al. used a sustainability and spread framework and KT strategies underpinned by this framework, adapting strategies such as training to continue over time [62]. Schnipper and colleagues refined their EBI and adapted their KT strategies for the scale and spread of an EBI. These changes were based on the results of a mixed-methods evaluation of the EBI post-implementation [57]. Sving et al. adapted their KT strategy of an external facilitator from the implementation to sustainability periods. After 2 years, the role of the external facilitator changed, whereby the facilitator provided support when requested from the unit. This study continued with quality improvement measures every month, a key strategy for the sustainability of the EBI [59]. Of the 25 included studies, only one study used a combination of adapted KT strategies from implementation and new KT sustainability strategies [67]. In this study, MacDonald et al. used the Dynamic Sustainability Framework (DSF) to adapt implementation strategies to sustainability strategies [67]. Four studies [61, 68, 69, 77] reported that they used the same KT strategies in the EBI implementation and sustainability periods, and one of these studies [68] stated that they demonstrated the sustainability of the EBI 5 years post-implementation with the continuation of KT strategies (audit and feedback, local champion). Kingsnorth et al.’s study reported planning for and considering sustainability from the beginning, using multidimensional KT strategies throughout, which included health electronic record revisions, audit and feedback built into the system, mentoring, and education [69]. Only two studies reported using a brand-new KT strategy specifically for EBI sustainability [75, 76]. Of these, one study introduced rapid improvement cycles as a strategy for the sustainability of the EBI, coined as ‘continuous improvement’ [75]. This phase occurred over a 2-year period, commencing immediately after the implementation phase [75]. The other study viewed EBI sustainability as dynamic and introduced new KT strategies based on EBI outcome results over a 5-year period [76].

**Table 6:** Use of KT strategies for EBI implementation and/or sustainability

Use of KT strategies	*(n = )*	Study reference
Brand new strategy for sustainability	2	[75, 76]
Adapted a KT strategy to use it for EBI sustainability	7	[53, 57, 59, 62, 64, 71, 74]
Combination of adapted and new KT strategies for EBI sustainability	1	[67]
Used the same strategies for EBI implementation and sustainability	4	[61, 68, 69, 77]
Did not report whether they used different or the same KT strategies between EBI implementation and sustainability	11	[51, 55, 56, 58, 60, 63, 65, 66, 70, 72, 73]

### Reported outcomes

Few studies reported on KT strategy outcomes. Five papers reported on the acceptability of 10 KT strategies used [55, 59, 68, 71, 74], 5 papers reported on the adoption of the KT strategies used [58, 60, 62, 69, 71], and 4 papers reported on the sustainability of 12 KT strategies used [51, 55, 67, 77]. For example, Lai et al. reported that the EBI champion role was readily accepted within the culture that exists in the clinical unit. Three of the five papers that reported on the adoption of a KT strategy were on education and training. For example, Willis et al. reported that the adoption of training as a KT strategy for EBI sustainability was a challenge due to inconsistent refresher training and poor coordination across the included clinical units [58]. Similarly, Santos et al. [65] reported poor adoption of training as a KT strategy for the sustainability of their selected EBI, noting scheduling difficulties. The four papers that reported on the sustainability of KT strategies used, focused on a variety of strategies. For example, Jaladanki [77] et al reported on the sustainability of five KT strategies (EBI champion, quality monitoring, EHR revision, ongoing reminders, and EBI multidisciplinary team), and MacDonald [67] et al, reported on four KT strategies (EBI champion, program facilitator, education, and leadership support). Education was one of the most reported KT strategies in relation to sustainability. For example, Mc Connell et al. [62] reported that the withdrawal of a dedicated facilitator meant that education was delivered informally to new staff by other staff members which led to frustration for staff who felt that formal education was key to successful implementation and sustainability.

The most reported sustainability outcome was the continuation of EBI activities or components (*n* = 24). The other most reported sustainability outcomes of the EBI were the continuation of benefits for staff and patients (*n* = 22). For example, Nkoy et al. [74] reported that they observed sustained reductions in asthma readmissions (*P* = .026) and length of stay (*P* = .001), a trend reduced costs (*P* = .094), and no change in hospital resource use, ICU transfers, or deaths. Another commonly reported sustainability outcome was the maintenance of new policies and procedures created because of the EBI (*n* = 15). For example, Becker et al. [57] reported that their EBI was declared an expected practice by nursing shared governance, supported by the nurse executive, and incorporated into the nursing strategic plan. Rutman et al. [76] reported on the maintenance of procedures for the management of acute gastroenteritis for children presenting to the ED over a 10-year period following the implementation of a clinical pathway using staff involvement, education, and reminders. Table 7 details the reported sustainability outcomes of the EBIs from the included studies.

**Table 7:** Reported sustainability outcomes of the EBIs from the included studies

Author	Benefits for patients, staff and stakeholders continue	EBI activities or component of the intervention continue	Maintenance of relationship, partnership, or networks	Maintenance of new procedures, and policies	Attention and awareness of the problem or issue is continued or increased	Replication Roll-out or scale-up of the EBI	Capacity built within staff, stakeholders, and communities continues	Adaptation in response to new evidence or contextual influences	Gaining further funds to continue the EBI and maintain improvement
Algaze [75]	✓								
Allen [74]	✓	✓	✓						
Andersson [73]	✓	✓		✓					
Barbieri [72]	✓	✓		✓					
Becker [70]	✓	✓	✓	✓	✓		✓	✓	
Comino-Sanz [70]	✓	✓		✓					
Erdei [76]	✓	✓	✓	✓	✓	✓	✓	✓	
Jaladanki [77]	✓	✓	✓	✓		✓	✓	✓	
Kingsnorth [69]	✓	✓	✓	✓	✓		✓	✓	
Lai [68]	✓	✓	✓						
MacDonald [67]	✓	✓	✓	✓		✓	✓	✓	
McConnell [62]		✓			✓		✓		
Moro [66]		✓							
Nkoy [53]	✓	✓	✓	✓		✓	✓	✓	
Oetgen [65]	✓	✓					✓	✓	
Parand [64]			✓	✓	✓		✓	✓	
Rutman [63]	✓	✓		✓				✓	
Santos [62]	✓	✓	✓	✓	✓	✓	✓	✓	✓
Schnipper [57]	✓	✓				✓		✓	
Shuman [61]	✓	✓							
Stevens [60]	✓	✓							
Storm-Versloot [51]	✓	✓	✓		✓				
Sving [59]	✓	✓		✓	✓		✓	✓	✓
Tamboli [56]	✓	✓	✓	✓		✓		✓	
Willis [58]	✓	✓	✓	✓		✓	✓	✓	
Totals:	22	23	13	15	8	8	12	14	2

## Discussion

The implementation of EBIs to improve healthcare does not always result in their long-term use or continued benefit for patients or health systems [33]. This gap illustrates the need to examine EBI sustainability as a separate phenomenon [34]. There has been a growing evidence base on factors that influence sustainability [78–80] and theoretical approaches to guide the sustainability of EBIs across various healthcare settings [48, 81]. More recently, there has been research conducted on KT strategies for sustaining public health EBIs [29]. However, there is no consolidated evidence on what and how KT strategies are used to support the ongoing use of EBIs across different institutional healthcare settings. This scoping review aimed to address this gap by synthesizing 25 studies reporting on KT strategies used to support the long-term sustainability of EBIs in healthcare institutional settings.

### What KT strategies are used to facilitate EBI sustainability

We identified training and education (*n* = 38) and the development of stakeholder interrelationships (*n* = 33) as the most reported ERIC KT strategy clusters employed to promote the sustainability of EBIs in our review. This finding is consistent with previous reviews of KT strategies for the implementation of guidelines and EBIs, as well as reviews on the sustainability of KT strategies in healthcare decision-making and public health settings [29, 31]. Future research should consider how education and training KT strategies, such as outreach visits, learning collaboratives, and presentations should be adapted to meet the evolving knowledge needs of the target audience from implementation to sustainability of an EBI.

Developing stakeholder relationships was the second most reported ERIC KT strategy cluster, which includes the use of opinion leaders, EBI champions, and multidisciplinary teams. This finding speaks to the relational nature of EBI implementation and sustainability [48]. Similarly, a previous review on EBI sustainability found that stakeholder participation was a determinant in 79% of theoretical approaches used for the sustainability efforts of EBIs across healthcare settings [48]. A recent study on factors influencing the sustainability and scale-up of a primary healthcare EBI also found that having a leader-champion, facilitation by local facilitators and researchers, and organizational and leadership support as strategies that facilitated sustainability [78].

### How are KT strategies used to sustain EBI?

Anticipating that knowledge needs may change over time, as the target audience becomes more familiar with the EBI, we believed that it was important to examine whether: (i) the same KT strategies were used for implementation and sustainability; (ii) new KT strategies were introduced for sustainability; or, (iii) adaptations were made to the KT strategies to support sustainability of the EBI. Our findings showed that 44% (*n* = 11/25) of studies did not report whether they used different or the same KT strategies between EBI implementation and sustainability. Furthermore, 28% (*n* = 7/25) of the included studies adopted a KT strategy used to support EBI sustainability [82]. Adaptation may mean changes to the design or delivery of the KT strategy during implementation and sustainability efforts [83, 84]. The papers included in our review provide valuable details on what and how KT strategies are used to support the sustainability of EBIs in healthcare institutions. Future studies should provide the description needed to understand how the KT strategies are adapted or not from implementation to sustainability efforts.

### Reported outcomes

Overall, from the included studies it was difficult to synthesize what KT strategies were most acceptable, feasible, appropriate, or adoptable for EBI sustainability across institutional healthcare settings. The studies that reported on KT strategy outcomes primarily focused on the acceptability and adoption of the KT strategies used. This is consistent with a recent review of implementation outcomes, which found that 52% of included studies examined acceptability, while penetration, sustainability, and cost were examined less frequently [85]. We echo Proctor et al.’s recommendations for a more objective measurement of KT strategy (implementation) outcomes to fully understand how KT strategies are being used to facilitate the sustainability of EBIs across different healthcare contexts [38].

Most included studies evaluated EBI sustainability outcomes related to the continuation of EBI activities and the continuation of benefits for staff and patients. This is like a review by Flynn et al. which found that 70% of their included studies evaluated continued benefits for patients, staff, and stakeholders and that the most frequently reported sustained benefits were improved health outcomes and improved quality of care [82]. Similar to our review, Flynn et al. also found that one of the least reported EBI sustainability outcomes was gaining further funds to continue the EBI and to maintain improvements [82]. Our findings also echo a review by Lennox et al., which found that only 21% of included studies reported any information on EBI sustainability outcomes in healthcare [79].

### Implications

First, this scoping review mapped what and how KT strategies are used for the sustainability of EBIs across institutional healthcare settings. Our review findings provide insights for health system leaders, decision-makers, and researchers about KT strategies being used to facilitate the sustainability of EBIs in institutional healthcare settings. Our findings suggest that health system leaders and decision-makers may want to pay special attention to the people involved, including champions and staff support, training and education, and evaluative and iterative strategies when aiming to sustain an EBI.

Second, this scoping review identified research gaps in the existing literature. To advance understanding of *how* to employ KT strategies for EBI sustainability, we recommend clearly reporting the dose, frequency, adaptations, fidelity, and cost of KT strategies. EBI sustainability literature has made important strides to enhance our understanding of KT strategies for sustainability, including the recent adaption, refinement, and extension of the ERIC taxonomy to include an explicit focus on sustainment [86]. We recommend using this revised taxonomy in future EBI implementation and sustainability efforts and include specific details such as KT strategy description, dose, frequency, and adaptations when first used to implement the EBI through to its use for the sustainability of the EBI. The use of reporting guidelines, such as the Standards for Reporting Implementation Studies (StaRI), AIMD Framework [47], and the TIDieR Checklist [84], but with clear reporting on if/how KT strategies are adapted for the sustainability of EBIs, could strengthen this evidence base [87].

Third, like Penno et al. [88], our work suggests that sustainability is a dynamic process with implementation and sustainability better conceptualized as a continuum rather than two discrete time periods or phases. This continuum conceptualization supports the consideration of how KT strategies for EBI sustainability over time differ from EBI implementation and/or potentially overlap. This continuum also supports the idea that over time the target audience may become more familiar with the EBI and as a result, KT strategies may need to be adapted.

Fourth, building on this scoping review, future research is needed that compares KT strategies in sustainability research, to determine how aspects of the EBI, potential adopters, and other context features interact with KT strategies to influence initial and ongoing EBI use. This will help determine which strategies are most pertinent to EBI sustainability. As such, efforts on KT ‘sustainability’ strategies are needed to add to this evidence base including experimental study designs to test different types of KT strategies, as well as other non-experimental designs to understand how, why, and under what contexts certain KT strategies work or not for EBI sustainability. We recommend the use of realist approaches to unpack the causal relationships between contexts (e.g., culture for change), the EBI (e.g., a clinical pathway), mechanisms of change (e.g., KT strategy) that lead to sustainability outcomes (e.g., continuation of benefits for patients). Such evidence would be highly beneficial to researchers, healthcare leaders, and decision-makers who need guidance on what KT strategy to select to support not only implementation but also EBI sustainability across different healthcare environments.

## Strengths and limitations

Findings from this scoping review should be considered with the following limitations in mind. We only included published studies in the English language and peer-reviewed primary studies in this review. We recognize that there is the possibility of relevant articles not being included in our search strategy. This review was descriptive in nature, given its scoping review focus. As such the study reports what strategies are being used but does not describe the effectiveness of the KT strategies. As such, our review findings are limited in their ability to make recommendations on the effectiveness of KT strategies for the sustainability of EBIs across institutional healthcare settings and the relationship between the EBI, KT strategy selected, and subsequent implementation and sustainability outcomes.

This review used a systematic approach to our search strategy and screening with multiple databases and employed theoretical frameworks/taxonomies/classification systems to understand how the findings align with other implementation and sustainability literature. Our review highlights that the implementation and sustainability of EBIs in healthcare is a continuum and the need for a better understanding of the potential extent of KT strategy adaptation from the initial implementation of an EBI to sustainability.

## Conclusion

It is only with the sustainability of EBIs that patient, provider, and health system outcomes will be realized, hence the imperative to better understand (how and to what extent) KT strategies support the ongoing use of EBIs. Our review has emphasized areas that require further research (e.g., KT strategy adaptation for EBI sustainability) and the need for reporting on KT strategies for the sustainability of EBIs in healthcare. Advancing our understanding in this area would facilitate better design, selection, tailored, and adapted use of KT strategies for EBI sustainability, thereby contributing to improved patient, provider, and health system outcomes.

### Supplementary Information

**Additional file 1.** Preferred Reporting Items for Systematic reviews and Meta-Analyses extension for Scoping Reviews (PRISMA-ScR) Checklist.

**Additional file 2.** Search strategies by database.

**Additional file 3.** The reported aims, ingredients, mechanism, and delivery of the KT strategies.

## Data Availability

The datasets used and/or analyzed during the current study are available from the corresponding author on reasonable request.

## Abbreviations

KT: Knowledge translation
EBIs: Evidence-based interventions
