# Supplementary material for: Knowledge translation strategies to support the sustainability of evidence-based interventions in healthcare: a scoping review
Source: Implement Sci. 2023 Dec 4;18:69. doi: 10.1186/s13012-023-01320-0 (PMC10694920; doi:10.1186/s13012-023-01320-0)
Supplement: Supplementary file 2 — Additional file 2. Search strategies by database. [file 13012_2023_1320_MOESM2_ESM.docx]

**Ovid MEDLINE(R) ALL <1946 to November 03, 2021>**

**Date of search: November 4th, 2021**

1 ((program* or initiative* or project*) adj2 sustain*).ti,kf. 419

2 (sustain* or institutionali* or routini* or normali?ation).ti. or ((sustain or sustainab* or sustaining or institutionali* or routini* or normali?ation) adj5 (program* or project? or service* or system? or quality or culture or evidence or assess* or measur* or evaluat* or indicator* or analys* or model* or framework* or theor* or approach* or change* or innovat*)).ab. 85829

3 ((longterm or "long-term" or longitudinal or continuing or continuous or enduring or lasting or "over time") adj3 (application* or adopt* or innovat* or change? or improvement* or outcome* or success*)).ti,ab. 189074

4 1 or 2 or 3 273375

5 *translational medical research/ or *diffusion of innovation/ or *quality improvement/ or *models, organizational/ or *health impact assessment/ or *program planning/ or *health services research/ 52090

6 ((translat* or implement* or adopt* or uptake or scale) and (change* or improv* or research or knowledge or information or innovat* or evidence or outcome*)).ti,ab,kf. 1265972

7 (((change or improv* or implement*) adj3 (manag* or strateg* or success*)) or (quality adj1 improv*)).ti,ab,kf. or (chang* or improv*).ti. 1029103

8 *Organizational Innovation/ or exp Health Services Accessibility/og, fs 16367

9 (((Knowledge or evidence or research or innovation or information) adj4 (translat* or transfer* or mobiliz* or mobilis* or exchange or implement* or disseminat* or utiliz* or utilis* or uptake or adopt* or communicat* or sharing or educat*)) or "research into practice" or "evidence into practice" or "knowledge to action" or "knowledge gap*" or "research to policy" or "translational science" or "implementation science" or "decision making" or "policy making").mp. 495341

10 5 or 6 or 7 or 8 or 9 2516764

11 ("health* innovation*" or "health care innovation*").ti,ab,kf. 984

12 exp Evidence-Based Practice/ 91965

13 ("evidence-based" or "evidence-informed" or EBP or EBM or EBI).ti,ab,kf. 145804

14 critical pathways/ 7291

15 clinical pathway?.ti,ab. 3907

16 exp Health Policy/ 111641

17 ((health* or "health care" or hospital* or nursing or medic* or surg* or patient* or care) adj4 (policy or policies)).ti,kf. 23955

18 clinical practice guideline?.ab. 12663

19 11 or 12 or 13 or 14 or 15 or 16 or 17 or 18 337660

20 (((health or patient*) adj2 care) or healthcare or hospital* or outpatient* or clinic* or "nursing home*" or "long term care" or LTC or "assisted living").ti,ab,kf. 6092743

21 ((primary or secondary or tertiary) adj2 (care or setting or healthcare)).ti,ab,kf. 235302

22 20 or 21 6151226

23 4 and 10 and 19 and 22 3414

24 animals/ or (veterinar* or zoo or zoos or species or habitat* or environment* or conservation or insect* or land management or "land use" or pollution or water or wastewater or soil or biodiesel or biofuel or urban or air quality or clean air or agricultur* or climate change or global warming or life sustaining or school* or (institutionali?ed adj3 (people or person* or patient* or elderly))).mp. 9813222

25 (((sustained or longterm or "long-term" or continuous or lasting) adj2 (release or attention or remission or sleep or control or response or decrease* or increase*)).ti. not (sustainab* or sustainment).mp.) or (sustain* adj3 (injur* or wound*)).mp. 26867

26 24 or 25 9833717

27 23 not 26 2855

28 limit 27 to (address or autobiography or bibliography or biography or clinical trials, veterinary as topic or comment or congress or consensus development conference or consensus development conference, nih or dataset or dictionary or directory or editorial or interactive tutorial or interview or lecture or legal case or legislation or letter or news or newspaper article or observational study, veterinary or periodical index or personal narrative or portrait or randomized controlled trial, veterinary or "research support, american recovery and reinvestment act" or research support, nih, extramural or research support, nih, intramural or research support, non us gov't or research support, us gov't, non phs or research support, us gov't, phs or video-audio media or webcast) 1003

29 27 not 28 1852

**OVID Embase <1974 to 2021 November 03>**

**Date of search: November 4th, 2021**

1 ((program* or initiative* or project*) adj2 sustain*).ti,kf. 404

2 (sustain* or institutionali* or routini* or normali?ation).ti. or ((sustain or sustainab* or sustaining or institutionali* or routini* or normali?ation) adj5 (program* or project? or service* or system? or quality or culture or evidence or assess* or measur* or evaluat* or indicator* or analys* or model* or framework* or theor* or approach* or change* or innovat*)).ab. 109057

3 ((longterm or "long-term" or longitudinal or continuing or continuous or enduring or lasting or "over time") adj3 (application* or adopt* or innovat* or change? or improvement* or outcome* or success*)).ti,ab. 281971

4 1 or 2 or 3 388681

5 *translational research/ 7327

6 diffusion of innovation.mp. 768

7 *total quality management/ 25235

8 *nonbiological model/ 5030

9 *health impact assessment/ 2055

10 *program development/ 5300

11 *health services research/ 10755

12 *health care access/ and organiz*.ab. 721

13 ((translat* or implement* or adopt* or uptake or scale) and (change* or improv* or research or knowledge or information or innovat* or evidence or outcome*)).ti,ab,kf. 1726075

14 (((change or improv* or implement*) adj3 (manag* or strateg* or success*)) or (quality adj1 improv*)).ti,ab,kf. or (chang* or improv*).ti. 1244127

15 organizational innovation*.mp. 332

16 (((Knowledge or evidence or research or innovation or information) adj4 (translat* or transfer* or mobiliz* or mobilis* or exchange or implement* or disseminat* or utiliz* or utilis* or uptake or adopt* or communicat* or sharing or educat*)) or "research into practice" or "evidence into practice" or "knowledge to action" or "knowledge gap*" or "research to policy" or "translational science" or "implementation science" or "decision making" or "policy making").ti,ab,kf. 500744

17 or/5-16 3093011

18 ("health* innovation*" or "health care innovation*").ti,ab,kf. 1265

19 exp *evidence based practice/ 83098

20 ("evidence-based" or "evidence-informed" or EBP or EBM or EBI).ti,ab,kf. 191965

21 ("critical path*" or "clinical path*").ti,ab. 34787

22 ((health* or "health care" or hospital* or nursing or medic* or surg* or patient* or care) adj4 (policy or policies)).ti,kf. 28258

23 clinical practice guideline?.ab. 18191

24 or/18-23 325093

25 (((health or patient*) adj2 care) or healthcare or hospital* or outpatient* or clinic* or "nursing home*" or "long term care" or LTC or "assisted living").ti,ab,kf. 8530081

26 ((primary or secondary or tertiary) adj2 (care or setting or healthcare)).ti,ab,kf. 331848

27 25 or 26 8605373

28 4 and 17 and 24 and 27 4092

29 (animal* or veterinar* or zoo or zoos or species or habitat* or environment* or conservation or insect* or land management or "land use" or pollution or water or wastewater or soil or biodiesel or biofuel or urban or air quality or clean air or agricultur* or climate change or global warming or life sustaining or school* or (institutionali?ed adj3 (people or person* or patient* or elderly))).mp. 9984682

30 (((sustained or longterm or "long-term" or continuous or lasting) adj2 (release or attention or remission or sleep or control or response or decrease* or increase*)).ti. not (sustainab* or sustainment).mp.) or (sustain* adj3 (injur* or wound*)).mp. 36179

31 29 or 30 10012151

32 28 not 31 3433

33 limit 32 to (books or chapter or conference abstract or conference paper or "conference review" or editorial or letter or note or short survey) 1244

34 32 not 33 2189

**CINAHL (1936 - present) via EBSCOhost**

**Date of search: November 4th, 2021**

**Results: 1335**

S1 TI ( ((program* or initiative* or project*) N2 sustain*) ) OR TI ( ((program* or initiative* or project*) N2 sustain*) ) OR AB ( ((sustain or sustainab* or sustaining or institutionali* or routini* or normali#ation) N5 (program* or project# or service* or system# or quality or culture or evidence or assess* or measur* or evaluat* or indicator* or analys* or model* or framework* or theor* or approach* or change* or innovat*)) )

S2 TI ( ((longterm or "long-term" or longitudinal or continuing or continuous or enduring or lasting or "over time") N3 (application* or adopt* or innovat* or change* or improvement* or outcome* or success*)) ) OR AB ( ((longterm or "long-term" or longitudinal or continuing or continuous or enduring or lasting or "over time") N3 (application* or adopt* or innovat* or change* or improvement* or outcome* or success*)) )

S3 S1 OR S2

S4 (MH "Translational Medical Research")

S5 (MH "Diffusion of Innovation+")

S6 (MM "Quality Improvement")

S7 (MM "Quality Management, Organizational")

S8 (MM "Health Impact Assessment")

S9 (MM "Program Development+")

S10 (MM "Health Services Research+")

S11 TI ( ((translat* or implement* or adopt* or uptake or scale) and (change* or improv* or research or knowledge or information or innovat* or evidence or outcome*)) ) OR AB ( ((translat* or implement* or adopt* or uptake or scale) and (change* or improv* or research or knowledge or information or innovat* or evidence or outcome*)) )

S12 TI ( (((change or improv* or implement*) N3 (manag* or strateg* or success*)) or (quality N1 improv*)) ) OR AB ( (((change or improv* or implement*) N3 (manag* or strateg* or success*)) or (quality N1 improv*)) )

S13 TI (chang* or improv*)

S14 TI ( (((Knowledge or evidence or research or innovation or information) N4 (translat* or transfer* or mobiliz* or mobilis* or exchange or implement* or disseminat* or utiliz* or utilis* or uptake or adopt* or communicat* or sharing or educat*)) or "research into practice" or "evidence into practice" or "knowledge to action" or "knowledge gap*" or "research to policy" or "translational science" or "implementation science" or "decision making" or "policy making") ) OR AB ( (((Knowledge or evidence ...

S15 S4 OR S5 OR S6 OR S7 OR S8 OR S9 OR S10 OR S11 OR S12 OR S13 OR S14

S16 TI ( ("health* innovation*" or "health care innovation*") ) OR AB ( ("health* innovation*" or "health care innovation*") )

S17 (MM "Professional Practice, Evidence-Based+")

S18 TI ( ("evidence-based" or "evidence-informed" or EBP or EBM or EBI) ) OR AB ( ("evidence-based" or "evidence-informed" or EBP or EBM or EBI) )

S19 (MH "Critical Path")

S20 TI "clinical pathway*" OR AB "clinical pathway*"

S21 TI ((health* or "health care" or hospital* or nursing or medic* or surg* or patient* or care) N4 (policy or policies))

S22 AB "clinical practice guideline*"

S23 S16 OR S17 OR S18 OR S19 OR S20 OR S21 OR S22

S24 TI ( (((health or patient*) N2 care) or healthcare or hospital* or outpatient* or clinic* or "nursing home*" or "long term care" or LTC or "assisted living") ) OR AB ( (((health or patient*) N2 care) or healthcare or hospital* or outpatient* or clinic* or "nursing home*" or "long term care" or LTC or "assisted living") )

S25 TI ( ((primary or secondary or tertiary) N2 (care or setting or healthcare)) ) OR AB ( ((primary or secondary or tertiary) N2 (care or setting or healthcare)) )

S26 S24 OR S25

S27 S3 AND S15 AND S23 AND S26

S28 (animal* or veterinar* or zoo or zoos or species or habitat* or environment* or conservation or insect* or "land management" or "land use" or pollution or water or wastewater or soil or biodiesel or biofuel or urban or "air quality" or "clean air" or agricultur* or "climate change" or "global warming" or "life sustaining" or school*)

S29 ( ((sustained or longterm or "long-term" or continuous or lasting) N2 (release or attention or remission or sleep or control or response or decrease* or increase*)) ) OR ( (sustain* N3 (injur* or wound*)) )

S30 S28 OR S29

S31 S27 AND S30

S32 S27 NOT S31

S33 S27 NOT S31 limit to scholarly peer-review journals

**Scopus via Elsevier (1976 - Present)**

**Date of search: November 4th, 2021**

**Results: 1901**

( ( ( TITLE ( ( ( program*  OR  initiative*  OR  project* )  W/2  sustain* ) ) )  OR  ( TITLE ( sustain*  OR  institutionali*  OR  routini*  OR  normali?ation ) )  OR  ( ABS ( ( sustain*  OR  institutionali*  OR  routini*  OR  normali?ation )  .ti.  OR  ( ( sustain  OR  sustainab*  OR  sustaining  OR  institutionali*  OR  routini*  OR  normali?ation )  W/5  ( program*  OR  project?  OR  service*  OR  system?  OR  quality  OR  culture  OR  evidence  OR  assess*  OR  measur*  OR  evaluat*  OR  indicator*  OR  analys*  OR  model*  OR  framework*  OR  theor*  OR  approach*  OR  change*  OR  innovat* ) ) ) )  OR  ( TITLE-ABS ( ( ( longterm  OR  "long-term"  OR  longitudinal  OR  continuing  OR  continuous  OR  enduring  OR  lasting  OR  "over time" )  W/3  ( application*  OR  adopt*  OR  innovat*  OR  change?  OR  improvement*  OR  outcome*  OR  success* ) ) ) ) )  AND  ( ( TITLE-ABS ( ( ( translat*  OR  implement*  OR  adopt*  OR  uptake  OR  scale )  AND  ( change*  OR  improv*  OR  research  OR  knowledge  OR  information  OR  innovat*  OR  evidence  OR  outcome* ) ) ) )  OR  ( TITLE-ABS ( ( ( ( change  OR  improv*  OR  implement* )  W/3  ( manag*  OR  strateg*  OR  success* ) )  OR  ( quality  W/1  improv* ) ) ) )  OR  ( TITLE ( chang*  OR  improv* ) )  OR  ( TITLE-ABS ( ( ( ( knowledge  OR  evidence  OR  research  OR  innovation  OR  information )  W/4  ( translat*  OR  transfer*  OR  mobiliz*  OR  mobilis*  OR  exchange  OR  implement*  OR  disseminat*  OR  utiliz*  OR  utilis*  OR  uptake  OR  adopt*  OR  communicat*  OR  sharing  OR  educat* ) )  OR  "research into practice"  OR  "evidence into practice"  OR  "knowledge to action"  OR  "knowledge gap*"  OR  "research to policy"  OR  "translational science"  OR  "implementation science"  OR  "decision making"  OR  "policy making" ) ) ) )  AND  ( ( TITLE-ABS ( ( "health* innovation*"  OR  "health care innovation*" ) ) )  OR  ( TITLE-ABS ( ( "evidence-based"  OR  "evidence-informed"  OR  ebp  OR  ebm  OR  ebi ) ) ) )  AND  ( TITLE-ABS ( ( ( health  OR  patient* )  W/2  care )  OR  healthcare  OR  hospital*  OR  outpatient*  OR  clinic*  OR  "nursing home*"  OR  "long term care"  OR  ltc  OR  "assisted living"  OR  ( ( primary  OR  secondary  OR  tertiary )  W/2  ( care  OR  setting  OR  healthcare ) ) ) ) )  AND NOT  ( TITLE-ABS ( ( animal*  OR  veterinar*  OR  zoo  OR  zoos  OR  species  OR  habitat*  OR  environment*  OR  conservation  OR  insect*  OR  "land management"  OR  "land use"  OR  pollution  OR  water  OR  wastewater  OR  soil  OR  biodiesel  OR  biofuel  OR  urban  OR  "air quality"  OR  "clean air"  OR  agricultur*  OR  "climate change"  OR  "global warming"  OR  "life sustaining"  OR  school*  OR  ( institutionali?ed  W/3  ( people  OR  person*  OR  patient*  OR  elderly ) ) ) ) )  AND  ( LIMIT-TO ( DOCTYPE ,  "ar" )  OR  LIMIT-TO ( DOCTYPE ,  "re" ) )

**Cochrane Library via Wiley (1993 - Present)**

**Date of search: November 4th, 2021**

#1 ((program* or initiative* or project*) NEAR/2 sustain*):ti 38

#2 (sustain* or institutionali* or routini* or normalization or normalisation):ti 6814

#3 ((sustain or sustainab* or sustaining or institutionali* or routini* or normali?ation) NEAR/5 (program* or project? or service* or system? or quality or culture or evidence or assess* or measur* or evaluat* or indicator* or analys* or model* or framework* or theor* or approach* or change* or innovat*)):ab 3250

#4 ((longterm or "long-term" or longitudinal or continuing or continuous or enduring or lasting or "over time") NEAR/3 (application* or adopt* or innovat* or change? or improvement* or outcome* or success*)):ti,ab 25999

#5 #1 or #2 or #3 or #4 35321

#6 ((translat* or implement* or adopt* or uptake or scale) and (change* or improv* or research or knowledge or information or innovat* or evidence or outcome*)):ti,ab 201731

#7 (((change or improv* or implement*) NEAR/3 (manag* or strateg* or success*)) or (quality NEAR/1 improv*)):ti,ab or (chang* or improv*):ti 110200

#8 (((Knowledge or evidence or research or innovation or information) NEAR/4 (translat* or transfer* or mobiliz* or mobilis* or exchange or implement* or disseminat* or utiliz* or utilis* or uptake or adopt* or communicat* or sharing or educat*)) or "research into practice" or "evidence into practice" or "knowledge to action" or "knowledge gap*" or "research to policy" or "translational science" or "implementation science" or "decision making" or "policy making"):ti,ab 26126

#9 #6 or #7 or #8 299440

#10 ("health* innovation" or "health care innovation"):ti,ab 34

#11 ("evidence-based" or "evidence-informed" or EBP or EBM or EBI):ti,ab 16354

#12 clinical pathway:ti,ab 350

#13 ((health* or "health care" or hospital* or nursing or medic* or surg* or patient* or care) NEAR/4 (policy or policies)):ti 181

#14 clinical practice guideline:ab 282

#15 #10 or #11 or #12 or #13 or #14 17090

#16 #5 and #9 and #15 682

#17 (((health or patient*) NEAR/2 care) or healthcare or hospital* or outpatient* or clinic* or "nursing home*" or "long term care" or LTC or "assisted living"):ti,ab 747362

#18 ((primary or secondary or tertiary) NEAR/2 (care or setting or healthcare)):ti,ab 33097

#19 #17 or #18 754420

#20 #16 and #19 533

#21 (animal* or veterinar* or zoo or zoos or species or habitat* or environment* or conservation or insect* or "land management" or "land use" or pollution or water or wastewater or soil or biodiesel or biofuel or urban or "air quality" or "clean air" or agricultur* or "climate change" or "global warming" or "life sustaining" or school* or (institutionali?ed NEAR/3 (people or person* or patient* or elderly))):ti,ab 112911

#22 ((sustained or longterm or "long-term" or continuous or lasting) NEAR/2 (release or attention or remission or sleep or control or response or decrease* or increase*)):ti or (sustain* NEAR/3 (injur* or wound*)):ti,ab 3669

#23 #21 or #22 116439

#24 #20 NOT #23 424
